# Vascular permeability in a human tumour xenograft: molecular charge dependence

**DOI:** 10.1054/bjoc.1999.1171

**Published:** 2000-04-03

**Authors:** M Dellian, F Yuan, V S Trubetskoy, V P Torchilin, R K Jain

**Affiliations:** Department of Otorhinolaryngology, University of Munich, Munich, D-81366, Germany; Department of Biomedical Engineering, Duke University, Durham, NC 27708, USA; Mirus Corporation, Madison, WI, USA; Department of Pharmaceutical Sciences, Northeastern University, Boston, MA, USA; Department of Radiation Oncology, Massachusetts General Hospital, Harvard Medical School, Boston, MA, 02114, USA

**Keywords:** tumour, vascular permeability, macromolecules, charge, drug delivery

## Abstract

Molecular charge is one of the main determinants of transvascular transport. There are, however, no data available on the effect of molecular charge on microvascular permeability of macromolecules in solid tumours. To this end, we measured tumour microvascular permeability to different proteins having similar size but different charge. Measurements were performed in the human colon adenocarcinoma LS174T transplanted in transparent dorsal skinfold chambers in severe combined immunodeficient (SCID) mice. Bovine serum albumin (BSA) and IgG were fluorescently labelled and were either cationized by conjugation with hexamethylenediamine or anionized by succinylation. The molecules were injected i.v. and the fluorescence in tumour tissue was quantified by intravital fluorescence microscopy. The fluorescence intensity and pharmacokinetic data were used to calculate the microvascular permeability. We found that tumour vascular permeability of cationized BSA (pI-range: 8.6–9.1) and IgG (pI: 8.6–9.3) was more than two-fold higher (4.25 and 4.65 × 10^−7^cm s^−1^) than that of the anionized BSA (pI ≈ 2.0) and IgG (pI: 3.0–3.9; 1.11 and 1.93 × 10^−7^cm s^−1^, respectively). Our results indicate that positively charged molecules extravasate faster in solid tumours compared to the similar-sized compounds with neutral or negative charges. However, the plasma clearance of cationic molecules was ∼2 × faster than that of anionic ones, indicating that the modification of proteins enhances drug delivery to normal organs as well. Therefore, caution should be exercised when such a strategy is used to improve drug and gene delivery to solid tumours. © 2000 Cancer Research Campaign


British Journal of Cancer (2000) **82**(9), 1513–1518
© 2000 Cancer Research Campaign
DOI: 10.1054/ bjoc.1999.1171, available online at http://www.idealibrary.com on IDEAL®

### Vascular permeability in a human tumour xenograft: molecular charge dependence

**M Dellian[1], F Yuan[2], VS Trubetskoy[3], VP Torchilin[4] and RK Jain[5]**

[1]Department of Otorhinolaryngology, University of Munich, D-81366 Munich, Germany; [2]Department of Biomedical Engineering, Duke University, Durham, NC 27708, USA; [3]Mirus Corporation, Madison, WI, USA; [4]Department of Pharmaceutical Sciences, Northeastern University, Boston, MA, USA; [5]Department of Radiation Oncology, Massachusetts General Hospital, Harvard Medical School, Boston, MA 02114, USA

**Summary** Molecular charge is one of the main determinants of transvascular transport. There are, however, no data available on the effect of molecular charge on microvascular permeability of macromolecules in solid tumours. To this end, we measured tumour microvascular permeability to different proteins having similar size but different charge. Measurements were performed in the human colon adenocarcinoma LS174T transplanted in transparent dorsal skinfold chambers in severe combined immunodeficient (SCID) mice. Bovine serum albumin (BSA) and IgG were fluorescently labelled and were either cationized by conjugation with hexamethylenediamine or anionized by succinylation. The molecules were injected i.v. and the fluorescence in tumour tissue was quantified by intravital fluorescence microscopy. The fluorescence intensity and pharmacokinetic data were used to calculate the microvascular permeability. We found that tumour vascular permeability of cationized BSA (pI-range: 8.6–9.1) and IgG (pI: 8.6–9.3) was more than two-fold higher (4.25 and $4.65 \times 10^{-7}$ cm $s^{-1}$) than that of the anionized BSA (pI ≈ 2.0) and IgG (pI: 3.0–3.9; 1.11 and $1.93 \times 10^{-7}$ cm $s^{-1}$, respectively). Our results indicate that positively charged molecules extravasate faster in solid tumours compared to the similar-sized compounds with neutral or negative charges. However, the plasma clearance of cationic molecules was ~2 × faster than that of anionic ones, indicating that the modification of proteins enhances drug delivery to normal organs as well. Therefore, caution should be exercised when such a strategy is used to improve drug and gene delivery to solid tumours. © 2000 Cancer Research Campaign

**Keywords:** tumour; vascular permeability; macromolecules; charge; drug delivery

Inadequate delivery of macromolecules limits the efficacy of systemic therapies of tumours with cytokines, monoclonal antibodies, and oligonucleotides (Presant et al, 1994; Jain, 1994; 1998). One of the major barriers to the delivery is the tumour microvessel wall. The permeability of the wall to macromolecules is heterogeneous in tumours: there exist regions where tumour vessels are nearly impermeable to these molecules, while other regions are much more leaky than normal vessels (Yuan et al, 1994). The leakiness of tumour vessels is presumably caused by the large pores (~ several hundred microns) in the vascular endothelium (Yuan et al, 1995), and discontinuity in the basement membrane (Bosman et al, 1985). Therefore, electrically neutral macromolecules and nanoparticles circulating in the blood accumulate preferentially in tumour tissues.

In addition to the size selectivity, the transvascular transport is influenced by the charge of molecules. The charge-dependence of the transvascular transport is especially important for gene delivery to tumours, using polycationic liposomes (Gao and Huang, 1995) or amino polymers (Goldman et al, 1997). These non-viral vectors have shown promising results in gene delivery across the cell membrane (Gao and Huang, 1995; Goldman et al, 1997). Recently, Thurston et al (1998) demonstrated that cationic liposomes are preferentially taken up by angiogenic tumour endothelium, mostly through vesicular organelles.

*Received 19 November 1998*
*Revised 14 December 1999*
*Accepted 20 December 1999*

*Correspondence to:* RK Jain

In normal tissues, the luminal endothelial membrane is negatively charged (Curry et al, 1987; Turner et al, 1983; Vehaskari et al, 1984; Deen et al, 1980; Baldwin et al, 1991; Dermietzel et al, 1983). Thus, it restricts the extravasation of anionic macromolecules, as has been demonstrated in cultured endothelial cell monolayer (Sahagun et al, 1990) and various normal tissues (Jain, 1997; Curry et al, 1987; Turner et al, 1983; Vehaskari et al, 1984; Deen et al, 1980; Baldwin et al, 1991; Dermietzel et al, 1983; Adamson et al, 1988; Barrowcliffe et al, 1990; Gandhi and Bell, 1992; Gilchrist and Parker, 1985; Haraldsson et al, 1983; Khaw et al, 1991; Leypoldt and Henderson, 1993; Michel and Phillips, 1985; Öjteg et al, 1987; Parker et al, 1985; Rasio and Goresky, 1985; Triguero et al, 1989). Adamson et al (1988) have demonstrated that the microvascular permeability to α-lactalbumin ($M_r$ = 14 176, net charge –10) is approximately 50% of that to ribonuclease ($M_r$ = 13 683, net charge +4), suggesting that the microvascular permeability to the positively charged molecules is higher than the permeability to the negative ones. The transport restriction of anionic macromolecules is crucial for maintaining a fluid homeostasis in the body, due to the osmotic effect (Curry, 1984). However, tumour vessels are significantly different from normal vessels. The role of molecular charge in the transport across tumour vessel wall is still unknown. To address this issue, we quantified tumour microvascular permeability of bovine serum albumin (BSA) and IgG, as well as their derivatives, on which different charge groups were covalently attached. The quantification was based on the intravital fluorescence microscopy, in combination with the human colon adenocarcinoma LS174T model implanted in the transparent dorsal skinfold chambers in severe combined immunodeficient (SCID) mice. The results of the present study may have important implications in designing vehicles for delivering genes and drugs to solid tumours.

### MATERIALS AND METHODS

### Animals and tumour model

Dorsal skinfold chambers bearing the LS174T tumour were prepared in male severe combined immunodeficient (SCID) mice as described earlier (Leunig et al, 1992). In brief, titanium chambers were implanted in the dorsal skin of mice (male, 6–8 weeks old, 25–35 g) under anaesthesia (75 mg ketamine hydrochloride and 25 mg xylazine $kg^{-1}$ body weight, subcutaneously). Two days later, 2 µl of a dense suspension of human colon carcinoma cells (approx. $2 \times 10^5$ cells in phosphate buffered saline) was inoculated onto the striated muscle layer of the subcutaneous tissue in the chambers. Experiments for measurement of permeability were performed 2 weeks after tumour cell implantation.

### Preparation of charge-modified BSA

Bovine serum albumin (A7030; Sigma Chemical Co, St. Louis, MO, USA) was first fluorescently labelled by conjugation with carboxytetramethylrhodamine succimidyl ester (C-1171; Molecular Probes, Eugene, OR, USA). Free dye was removed on a gel filtration column (Econo-Pac 10DG; Bio-Rad Laboratories, Herculaes, CA, USA) equilibrated with 50 mM phosphate buffered saline (PBS; Sigma) containing 0.002% sodium azide (Sigma). This procedure yielded a molar dye/protein ratio of 1.3. Subsequently, aliquots of the BSA solution were anionized by succinylation, or cationized by conjugation with hexamethylenediamine. For succinylation (Klotz, 1967; Rennke and Venkatchalam, 1978), 10 mg of succinic anhydride (Sigma) in 50 ml of dimethylsulfoxide (DMSO, Sigma) was added dropwise in small increments to the solution of 10 mg protein in 1 ml of 0.2 M bicarbonate buffer, pH 8.0. During the incubation time (30 min at room temperature) pH was maintained at 8.0–8.5. For cationization (Triguero et al, 1989; Kumagai et al, 1987; Hoare and Koshland, 1967), free carboxylic groups of the protein (10 mg in 1 ml of 5 mM MES, pH 5.3, if necessary pH was maintained at 5.3 with 1 M HCl) were activated with 1-ethyl-3-(3-dimetlaminopropyl)carbodiimide (CDI, Sigma). For this purpose, two portions of CDI (10 mg in 100 ml water) were added to the protein solution with a 10 min interval. Immediately after the addition of the second portion of CDI, the activated protein was added upon stirring into 2 ml of 2 M hexamethylenediamine (Sigma) in water. pH of the solution was adjusted to 6.8 and the mixture was incubated for 5 h at room temperature. After conjugation, the products were purified by overnight dialysis, and high molecular weight aggregates were removed by elution through a Sepharose CL-6B column (Pharmacia). The major protein peak was pooled and stored at 4°C.

### Preparation of charge modified IgG

The solvent of mouse monoclonal IgG antibody (MOPC21; M-9269, Sigma) was exchanged to 0.2 M sodium bicarbonate buffer on a gel filtration column (Econo-Pac 10DG; Bio-Rad) and the solution concentrated to 1 mg protein $ml^{-1}$ by centrifugal filtration (Ultrafree-CL; Millipore, Bedford, MA, USA). pH was adjusted to 9.3. For fluorescent labelling, Cyanine 3 monofunctional dye (Cy3-Mono-OSu) (PA13104; Amersham Life Science, Arlington Heights, IL, USA) was used to yield high fluorescence intensity, which allowed in vivo experiments with small amounts of IgG (0.5 mg IgG $animal^{-1}$). Cy3-Mono-OSu was added at a ratio of 2 mg dye 10 mg $protein^{-1}$ and the solution stirred at room temperature for 60 min. Free dye was removed on a gel filtration column (Econo-Pac 10DG; Bio-Rad) equilibrated with 50 mM PBS containing 0.002% sodium azide (Sigma), and the solution concentrated to 1.8 mg protein $ml^{-1}$ by centrifugal filtration. Subsequently, anionic and cationic derivatives of IgG were prepared as described above for BSA.

### Measurement of molecular charge and weight

We did not quantify the net charge per molecule, since it depends on local tissue environment, especially extracellular pH which is heterogeneous in solid tumours (Helmlinger et al, 1997). Therefore, only the fraction of free lysine amino-groups in proteins that were modified with charge-bearing groups was quantified, using trinitrobenzenesulphonic acid titration method (Fields, 1971). We found that approximately 25–35% of all amino-groups accessible for modification was modified with charge-bearing groups. In addition, isoelectric points of the proteins were determined by isoelectric focusing using polyacrylamide gel slabs on a vertical electrofocusing apparatus. pI was quantified by comparison with protein standards (Bio-Rad) after Coomassie Blue staining of the gels. The proteins' molecular weight was analysed by SDS-PAGE (Mini-Protean II; Bio-Rad) with and without reducing agent β-mercaptoethanol in the sample buffer. Molecular size of native and charge-modified proteins was estimated by HPLC on analytical gel filtration column (Shodex Protein KW 804; Waters, Milford, USA) with precolumn (Shodex Protein WS 800P) using Waters 600E system controller and 996 photodiode array detector. Mobile phase was 0.05 M phosphate buffer at pH 7.4 and flow rate was 0.7 ml $min^{-1}$. We used three standard molecules: chymotrypsin (MW 23 kDa), amylase (MW 200 kDa), and thyroglobulin (MW 669 kDa), and found that positive, negative and native BSA and IgG gave indistinguishable peaks. All BSAs showed the same peak not very far from chymotrypsin, while all IgGs showed the same peaks close to amylase. These data indicate that charge modification did not significantly change the size of molecules.

### Measurement of tumour microvascular permeability

Effective microvascular permeability of the different proteins was measured using fluorescence microscopy as reported previously (Yuan et al, 1993; 1995). In brief, animals were anaesthetized as described above and immobilized in a polycarbonate tube on the microscope stage. The fluorescently labelled protein was dissolved in PBS and injected into the tail vein as a bolus (0.1 ml 25 g body $weight^{-1}$). Fluorescence intensity of tumour tissue was monitored by a 20× objective of a fluorescence microscope (Axioplan, Zeiss), and quantified over a period of 20 min by a photomultipier. We have quantified S/V in different tumours of the same cell line (LS174T) and found that S/V = 239 ± 82 $cm^2$ $cm^{-3}$ (Yuan et al, 1993). Therefore, we used the mean value of S/V in the estimation of microvascular permeability. In separate experiments using three animals per protein, the time constant of plasma clearance (K) was quantified through collecting arterial blood

 *© 2000 Cancer Research Campaign*

**Table 1** Characteristics of tracer molecules and their microvascular permeability in tumours. Anionized and cationized BSA and IgG were prepared from the native proteins as described in Materials and methods. **P* < 0.01 vs the corresponding anionized compound (Mann-Whitney U test)

| Name | $M_r$ | pI[a] (range) | Median K[a] (range) (100s) | Median $P_v$[a] (range) ($10^{-7}$ cm $s^{-1}$) |
|---|---|---|---|---|
| Native BSA[b] | 66 000 | 4.5 | 80.5 (77–130) | 1.61 (0.65–1.93) |
| Anionized BSA | 64 000 | ≈2.0 | 40.8 (37–45) | 1.11 (0.95–2.38) |
| Cationized BSA | 64 000 | 8.6–9.1 | 12.7 (12–13) | 4.25* (3.57–5.34) |
| Native IgG[b] | 160 000 | 6.0 | 50.0 (34–82) | 2.82 (1.47–4.07) |
| Anionized IgG | 155 000 | 3.0–3.9 | 39.3 (37–44) | 1.93 (1.11–2.53) |
| Cationized IgG | 155 000 | 8.6–9.3 | 11.0 (5–15) | 4.65* (4.25–5.27) |

[a]pI, isoelectric point; K, time constant of concentration decay in the plasma; $P_v$, microvascular permeability. [b]Data from Yuan et al (1995).

samples at t = 0, 1, 3, 5, 10 and 30 min following injection of the proteins, and fitting these data to an exponential function. Tumour microvascular permeability from experiments with 6–7 individual tumours for each of the different proteins, was calculated from these data according to Yuan et al (1993). Results for microvascular permeability are given as range or median ± standard error of median. Mann-Whitney U test was used to compare the difference in tumour microvascular permeability between the altered proteins.

### Mathematical modeling of tumour uptake

Electrical charge of molecules affects both transvascular transport and plasma clearance of drugs. Therefore, the amount of drug accumulation in solid tumours depends on the balance between these two processes. In this study, a two-compartmental model was used to investigate the effect of charge manipulation on the total accumulation of drugs in tumours. The governin gequation of the average concentration of drugs in tumours (C) is as follows:

$$\frac{dC}{dt} = \frac{PS}{V}(C_P - C) \qquad (1)$$

$$C_P = C_{P0}e^{-\frac{t}{K}}$$

where P is the vascular permeability, S/V is the vascular surface area per unit tissue volume, t is the time, $C_{P0}$ is the plasma concentration ($C_P$) immediately after intravenous injection, and K is the time constant of plasma clearance. We assumed that the initial concentration in tumours was zero, thus the solution of equation 1 is:

$$\frac{C}{C_{P0}} = \frac{(PS/V)K}{1-(PS/V)K}\left(e^{\frac{-PS}{V}t} - e^{\frac{-1}{K}t}\right) \qquad (2)$$

We further assumed that S/V = 239 $cm^2$ $cm^{-3}$ (Yuan et al, 1993). The values of K and P used in the simulation were measured directly in this study (see Table 1).

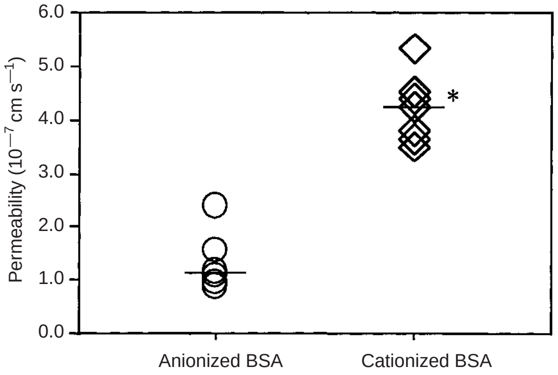

**Figure 1** Microvascular permeability of tumours to anionized (*n* = 6 tumours) and cationized BSA (*n* = 7 tumours). Bars depict median values of the groups. **P* < 0.01 (Mann-Whitney U test)

### RESULTS

Fluorescence microscopy revealed that the different proteins extravasated homogeneously along the vessel walls. Tumour microvascular permeability ($P_V$) to anionized and cationized BSA is shown in Figure 1. Permeability values were low for negatively charged BSA ($P_V$ = 1.11 ± 0.44 × $10^{-7}$ cm $s^{-1}$; median ± standard error of median), and almost fourfold higher to positively charged BSA ($P_V$ = 4.25 ± 0.26 × $10^{-7}$ cm $s^{-1}$). Characteristics of tracer molecules are given in Table 1.

The microvascular permeability in tumours of anionized and cationized IgG was slightly above values measured for corresponding BSA samples (Figure 2, Table 1). Similar to the data for charge-modified BSA, cationized IgG revealed highest permeability ($P_v$ = 4.65 ± 0.29 × $10^{-7}$ cm $s^{-1}$), and permeability of anionized IgG ($P_V$ = 1.93 ± 0.41 × $10^{-7}$ cm $s^{-1}$) was below the value measured for the native protein.

The plasma clearance rate revealed a similar behaviour for both proteins (Table 1). The time constant of the plasma clearance (K) was highest for the native proteins, slightly lower for the anionized compounds, and significantly lower for cationized BSA and IgG. These results are consistent with the data in the literature (Deen *et al*, 1980).

*© 2000 Cancer Research Campaign*

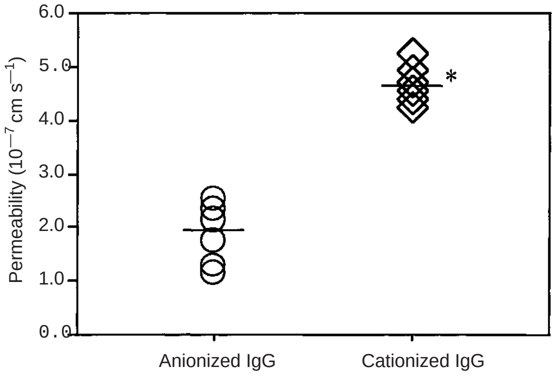

**Figure 2** Tumour microvascular permeability to anionized ($n$ = 6 tumours) and cationized IgG ($n$ = 6 tumours). Bars depict median values of the groups. *$P$ < 0.01 (Mann-Whitney U test)

The effect of charge on the accumulation of molecules in tumours was investigated, based on the mathematical simulation of average concentrations. We found that charge modification of BSA and IgG decreased the total accumulation of drugs (Figure 3A), through either reduction in microvascular permeability or increase in the plasma clearance. The total accumulation in tumours could be increased through cationization of drugs, if the plasma concentration was maintained at a constant level, i.e. the time constant of plasma clearance, $K \rightarrow \infty$ (Figure 3B). In this case, the accumulation was affected only by the microvascular permeability.

### DISCUSSION

### Molecular charge-dependence of vascular permeability

In comparison to tumour microvascular permeability of native BSA and native IgG (Table 1, Yuan et al, 1995), the permeability values were lower for negatively charged BSA and IgG, and more than twofold higher for the positively charged proteins. The charge-dependence in vascular permeability in tumours is consistent with observations in normal tissues (Jain, 1997; Curry *et al*, 1987; Turner et al, 1983; Vehaskari et al, 1984; Deen et al, 1980; Baldwin et al, 1991; Dermietzel et al, 1983; Adamson et al, 1988; Barrowcliffe et al, 1990; Gandhi and Bell, 1992; Gilchrist and Parker, 1985; Haraldsson et al, 1983; Khaw et al, 1991; Leypoldt and Henderson, 1993; Michel and Phillips, 1985; Öjteg et al, 1987; Parker et al, 1985; Rasio and Goresky, 1985; Triguero et al, 1989), suggesting the luminal surface of tumour vascular endothelium is negatively charged as well.

The effect of molecular charge on microvascular permeability has been studied in several organs including the mesentery (Adamson et al, 1988), the kidney (Rennke and Venkatchalam, 1978), the lung (Gilchrist and Parker, 1985) and the brain (Kumagai et al, 1987). On the one hand, negatively charged glycoproteins, coated on the luminal side of the endothelium known as glycocalyx in various normal organs (Turner et al, 1983; Vink and Duling, 1996) form a physiological barrier for the transcapillary movement of negatively charged molecules. Vehaskari et al (1984) have demonstrated that intravenous injection of polycations causes a significant increase in haematocrit and decrease in plasma albumin in nephrectomized rats. These results suggest that the

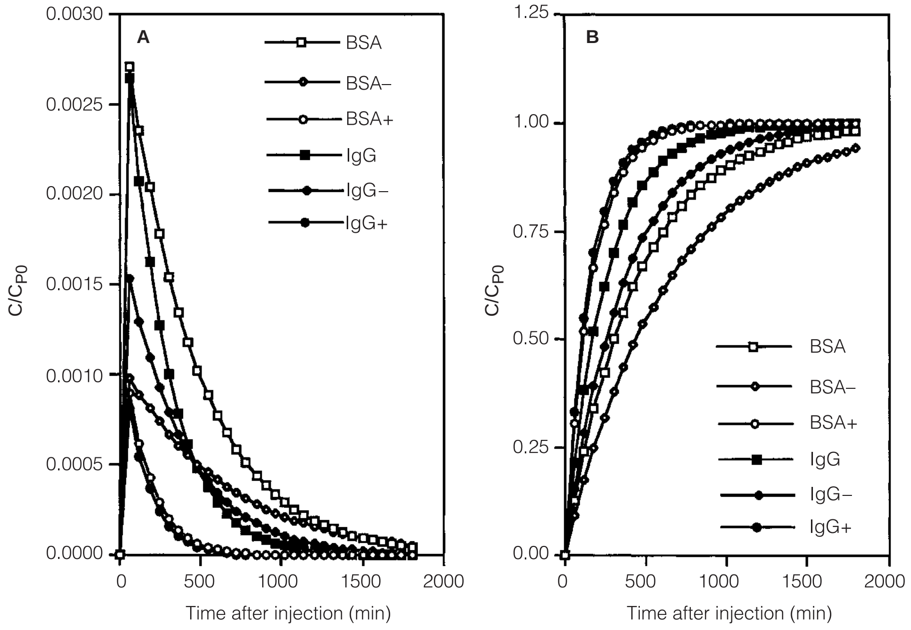

**Figure 3** Mathematical simulation of the average concentration (C) of BSA and IgG in a solid tumour as a function of time. (**A**) Simulation of the relation between tumour concentration and plasma concentration ($C/C_{PO}$) for the different proteins based on our experimental results following bolus injection. (**B**) Simulation of the relation between tumour concentration and plasma concentration ($C/C_{PO}$) for the different proteins, if the plasma concentration was maintained at a constant level: '+' and '–' indicate anionic or cationic modification of molecules

*© 2000 Cancer Research Campaign*

neutralization of the negative charge sites on the vessel wall can abolish the barrier effect of the glycocalyx layer on the transport of anionic macromolecules. On the other hand, the negatively charged endothelial surface may facilitate interactions between cationic molecules and the vascular endothelium. Kumagai et al (1987) observed a rapid binding and endocytosis of cationized albumin to isolated brain capillaries, suggesting that the anionic barrier facilitates the endothelial absorption and endocytosis of cationic molecules.

Cationic liposomes have been demonstrated to be taken up by endothelial cells in an organ-specific pattern with highest accumulation in the lung (McLean et al, 1997). However, angiogenic endothelial cells in tumours and in chronic inflammation revealed a preferential uptake of cationic liposomes, with a high proportion being associated with endothelial fenestrae (Thurston et al, 1998). Endothelial fenestrae are very frequently found on tumour endothelium (Roberts and Palade, 1997; Hobbs et al, 1998), and may thus be the site of extravasation of cationic proteins.

The absorption of cationic molecules to the endothelium and their subsequent internalization may potentially affect the vascular permeability measurement, since most techniques used are based on the quantification of the total accumulation of tracers in the vicinity of vessels. Therefore, the apparent vascular permeability data of cationic molecules accounts for the combined effect of the endothelial uptake (bound plus internalized) and the extravasation. The exact contributions of individual mechanisms to the apparent vascular permeability cannot be determined separately. However, we did not observe a significant staining of tumour vessels with the fluorescent tracers, indicating that the amount of bound proteins was insignificant compared to the extravasated molecules. The ratio of the bound versus extravasated macromolecules should be much lower in tumours than normal tissues, since tumour vascular endothelium is leaky (Yuan et al, 1993; 1995; Wu et al, 1993; Gerlowski and Jain, 1986; Dvorak et al, 1995) and the basement membrane is incomplete (Hobbs et al, 1998). Therefore, the effect of the endothelial uptake on the permeability measurement, if any, may be less important in tumours.

### Plasma clearance of charged macromolecules

The mechanism of the plasma clearance of macromolecules is different from that of small molecules. The cut-off size of renally filterable proteins and peptides is approximately 6.3 nm ($M_r$ ~ 60 000) (Behr et al, 1995), which is slightly smaller than the size of albumin. For dextran, this size is increased to approximately 8 nm (Deen et al, 1980), presumably due to the difference in the molecular configuration. Consequently, the excretion of proteins larger than the renally filterable size may initially require catabolism in the liver; and smaller metabolites can then be filtered and excreted through the kidney (Behr et al, 1995). In addition to liver and kidney, organs such as the lung (McLean et al, 1997) and the immune system may be involved in the clearance of charge modified molecules. Bass et al (1990) have shown that cationization of albumin alters its molecular conformation. The conformation change may increase the immunogenicity of exogenous molecules, in part due to an increased uptake by antigen-presenting cells (Apple et al, 1988). The immune response can cause vascular leakiness and significantly reduce the plasma half-life of molecules (Yamamoto et al, 1986; Adamski et al, 1987). However, such a response was unlikely in our experiments, since the permeability was quantified within 1 h after the intravenous injection of proteins. Taken together, we hypothesize that the charge-dependence of the plasma clearance soon after the injection depends on three effects: microvascular permeability in various normal tissues, catabolism of the macromolecules in the liver, and the clearance of their metabolites in the plasma through the kidney.

### Implications for drug and gene delivery to solid tumours

In conclusion, positively charged macromolecules extravasate faster in solid tumours compared to similar sized compounds with neutral or negative charges, suggesting that cationization may enhance delivery of therapeutic agents to solid tumours. However, the rapid clearance of cationic molecules from the plasma indicates that the charge modification enhances the drug delivery to normal organs as well. Therefore, caution should be exercised when such a strategy is used to improve the drug and gene delivery to solid tumours. This issue is especially important for gene therapy where polycationic liposomes and amino polymers are used as the delivering vectors. In general, the tumour specificity versus the enhanced delivery of drugs and genes has to be balanced in the development and evaluation of charge-modified drugs or vectors.

### ACKNOWLEDGEMENTS

We thank Yi Chen and Julia Kahn for excellent chamber preparations. This work was supported in part by National Cancer Institute Outstanding Investigator Grant R35-CA-56591 (to R.K.J.); M.D. was recipient of a Feodor-Lynen Fellowship from the Alexander von Humboldt-Foundation (1993–1995). Preliminary results of this work were presented at the 1996 Annual Meetings of the Microcirculatory Society in Bethesda, MD and Experimental Biology in Washington, DC.

### REFERENCES

Adamski SW, Langone JJ and Grega GJ (1987) Modulation of macromolecular permeability by immune-complexes and a beta-adrenoceptor stimulant. *Am J Physiol* **253**: H1586–H1595

Adamson RH, Huxley VH and Curry FE (1988) Single capillary permeability to proteins having similar size but different charge. *Am J Physiol* **254**: H304–H312

Apple RJ, Domen PL, Muckerheide A and Michael JG (1988) Cationization of protein agents. IV. Increased antigen uptake by antigen-presenting cells. *J Immunol* **140**: 3290–3295

Baldwin AL, Wu NZ and Stein DL (1991) Endothelial surface charge of intestinal mucosal capillaries and its modulation by dextran. *Microvasc Res* **42**: 160–178

Barrowcliffe MP, Zanelli GD, Ellison D and Jones JG (1990) Clearance of charged and uncharged dextrans from normal and injured lungs. *J Appl Physiol* **68**: 341–347

Bass PS, Drake AF, Wang Y, Thomas JH and Davies DR (1990) Cationization of bovine serum albumin alters its conformation as well as its charge. *Lab Invest* **62**: 185–188

Behr TM, Sharkey RM, Juweid ME, Blumenthal RD, Dunn RM, Griffiths GL, Bair HJ, Wolf FG, Becker WS and Goldenberg DM (1995) Reduction of the renal uptake of radiolabeled monoclonal antibody fragments by cationic amino acids and their derivatives. *Cancer Res* **55**: 3825–3834

Bosman FT, Havenith M and Cleutjens JP (1985) Basement membranes in cancer. *Ultrastruct Pathol* **8**: 291–304

Curry FE (1984) Mechanisms and thermodynamics of transcapillary exchange. In: *Handbook of Physiology*, Section 2, The Cardiovascular System, Vol. IV, *The Microcirculation*, Renkin EM and Michel CC (Eds) pp. 309–374. American Physiological Society: Bethesda

© 2000 Cancer Research Campaign 

Curry FE, Rutledge JC and Lenz JF (1987) Modulation of microvessel wall charge by plasma glycoprotein orosomucoid. *Am J Physiol* **257**: H1354–H1359

Deen WM, Satvat B and Jamieson JM (1980) Theoretical model for glomerular filtration of charged solutes. *Am J Physiol* **238**: F126–F139

Dermietzel R, Thurauf N and Kalweit P (1983) Surface charges associated with fenestrated brain capillaries. II. In vivo studies on the role of molecular charge in endothelial permeability. *Ultrastruct Res* **84**: 111–119

Dvorak HF, Brown LF, Detmar M and Dvorak AM (1995) Vascular permeability factor/vascular endothelial growth factor, microvascular hyperpermeability, and angiogenesis. *Am J Pathol* **146**: 1029–1039

Fields R (1971) The measurement of amino groups in proteins and peptides. *Biochem J* **124**: 581–590

Gandhi RR and Bell DR (1992) Importance of charge on transvascular albumin transport in skin and skeletal muscle. *Am J Physiol* **262**: H999–H1008

Gao X and Huang L (1995) Cationic liposome-mediated gene transfer. *Gene Ther* **2**: 710–722

Gerlowski LE and Jain RK (1986) Microvascular permeability of normal and neoplastic tissues. *Microvasc Res* **31**: 288–305

Gilchrist SA and Parker JC (1985) Exclusion of charged macromolecules in the pulmonary interstitium. *Microvasc Res* **30**: 88–98

Goldman CK, Soroceanu L, Smith N, Gillespie GY, Shaw W, Burgess S, Bilbao G and Curiel DT (1997) In vitro and in vivo gene delivery mediated by a synthetic polycationic amino polymer. *Nat Biotech* **15**: 462–466

Haraldsson B, Ekholm C and Rippe B (1983) Importance of molecular charge for the passage of endogenous macromolecules across continuous capillary walls, studied by serum clearance of lactate dehydrogenase (LDH) isoenzymes. *Acta Physiol Scand* **117**: 123–130

Helmlinger G, Yuan F, Dellian M and Jain RK (1997) Interstitial pH and $pO_2$ gradients in solid tumours in vivo: High-resolution measurements reveal a lack of correlation. *Nat Med* **3**: 177–182

Hoare DG and Koshland DE (1967) Method for the quantitative modification and estimation of carboxylic acid groups in proteins. *J Biol Chem* **242**: 2447–2453

Hobbs SK, Monsky WL, Yuan F, Roberts WG, Griffith L, Torchilin VP and Jain RK (1998) Regulation of transport pathways in tumour vessels: role of tumour type and microenvironment. *Proc Natl Acad Sci USA* **95**: 4607–4612

Jain RK (1994) Barriers to drug delivery in solid tumours. *Sci Am* **271**: 58–65

Jain RK (1997) 1996 Landis award lecture: Delivery of molecular and cellular medicine to solid tumours. *Microcirculation* **4**: 1–23

Jain RK (1998) The next frontier of molecular medicine: delivery of therapeutics. *Nat Med* **4**: 655–657

Khaw B, Klibanov A, O’donnell SM, Saito T, Nossiff N, Slinkin MA, Newell JB, Strauss HW and Torchilin VP (1991) Gamma imaging with negatively charge-modified monoclonal antibody: modification with synthetic polymers. *J Nucl Med* **32**: 1742–1751

Klotz IM (1967) Succinylation. In: *Methods in Enzymology*, Hirs CA (Ed) pp. 576–580. Academic Press: New York

Kumagai AK, Eisenberg JB and Pardridge WM (1987) Absorptive-mediated endocytosis of cationized albumin and a beta-endorphin-cationized albumin chimeric peptide by isolated brain capillaries: model system for blood–brain barrier transport. *J Biol Chem* **262**: 15214–15219

Leunig M, Yuan F, Menger MD, Boucher Y, Goetz AE, Messmer K and Jain RK (1992) Angiogenesis, microvascular architecture, microhemodynamics, and interstitial fluid pressure during early growth of human adenocarcinoma LS174T in SCID mice. *Cancer Res* **52**: 6553–6560

Leypoldt JK and Henderson LW (1993) Molecular charge influences transperitoneal macromolecule transport. *Kidney Int* **43**: 837–844

Mclean JW, Fox EA, Baluk P, Bolton PB, Haskell A, Pearlman R, Thurston G, Umemoto EY and Mcdonald DM (1997) Organ-specific endothelial cell uptake of cationic liposome-DNA complexes in mice. *Am J Physiol* **273**: H387–H404

Michel CC and Phillips ME (1985) The effects of bovine serum albumin and a form of cationised ferritin upon the molecular selectivity of the walls of single frog capillaries. *Microvasc Res* **29**: 190–203

Öjteg G, Nygren K and Wolgast M (1987) Permeability of renal capillaries. II. Transport of neutral and charged protein molecular probes. *Acta Physiol Scand* **129**: 287–294

Parker JC, Gilchrist S and Cartledge JT (1985) Plasma-lymph exchange and interstitial distribution volumes of charged macromolecules in the lung. *J Appl Physiol* **59**: 1128–1136

Presant CA, Wolf W, Waluch V, Wisemann C, Kennedy P, Blayney D and Brechner RR (1994) Association of intratumoural pharmacokinetics of fluorouracil with clinical response. *Lancet* **343**: 1184–1187

Rasio EA and Goresky CA (1985) Passage of ions and dextran molecules across the rete mirabile of the eel. The effects of charge. *Circ Res* **57**: 74–83

Rennke HG and Venkatchalam MA (1978) Glomerular permeability of macromolecules: effect of molecular configuration on the fractional clearance of uncharged dextran and neutral horseradish peroxidase in the rat. *Kidney Int* **13**: 278–288

Roberts WG and Palade GE (1997) Neovasculature induced by vascular endothelial growth factor is fenestrated. *Cancer Res* **57**: 765–772

Sahagun G, Moore SA and Hart MN (1990) Permeability of neutral vs. anionic dextrans in cultured brain microvascular endothelium. *Am J Physiol* **259**: H162–H166

Thurston G, Mclean JW, Rizen M, Baluk P, Haskell A, Murphy TJ, Hanahan D and Mcdonald DM (1998) Cationic liposomes target angiogenic endothelial cells in tumours and chronic inflammation in mice. *J Clin Invest* **101**: 1401–1413

Triguero D, Buciak JB, Yang J and Pardridge WM (1989) Blood–brain barrier transport of cationized immunoglobulin G: enhanced delivery compared to native protein. *Proc Natl Acad Sci USA* **86**: 4761–4765

Turner MR, Clough G and Michel CC (1983) The effects of cationised ferritin and native ferritin upon the filtration coefficient of single frog capillaries. Evidence that proteins in the endothelial cell coat influence permeability. *Microvasc Res* **25**: 205–222

Vehaskari VM, Chang CT, Stevens JK and Robson AM (1984) The effects of polycations on vascular permeability in the rat. A proposed role for charge sites. *J Clin Invest* **73**: 1053–1061

Vink H and Duling BR (1996) Identification of distinct luminal domains for macromolecules, erythrocytes, and leukocytes within mammalian capillaries. *Circ Res* **79**: 581–589

Wu NZ, Klitzman B, Rosner G, Needham D and Dewhirst MW (1993) Measurement of material extravasation in microvascular networks using fluorescence video-microscopy. *Microvasc Res* **46**: 231–253

Yamamoto A, Utsumi E, Sakane T, Hamaura T, Nakamura J, Hashida M and Sezaki H (1986) Immunological control of drug absorption from the gastrointestinal tract: the mechanism whereby intestinal anaphylaxis interferes with the intestinal absorption of bromthymol blue in the rat. *J Pharm Pharmacol* **38**: 357–362

Yuan F, Leunig M, Berk DA and Jain RK (1993) Microvascular permeability of albumin, vascular surface area, and vascular volume measured in human adenocarcinoma LS174T using dorsal chamber in SCID mice. *Microvasc Res* **45**: 269–289

Yuan F, Leunig M, Huang SK, Berk DA, Papahadjopoulos D and Jain RK (1994) Microvascular permeability and interstitial penetration of sterically stabilized (stealth) liposomes in a human tumour xenograft. *Cancer Res* **54**: 3352–3356

Yuan F, Dellian M, Fukumura D, Leunig M, Berk DA, Torchilin VP and Jain RK (1995) Vascular permeability in a human tumour xenograft: molecular size dependence and cutoff size. *Cancer Res* **55**: 3752–3756

 *© 2000 Cancer Research Campaign*
